# A Microalbuminuria Threshold to Predict the Risk for the Development of Diabetic Retinopathy in Type 2 Diabetes Mellitus Patients

**DOI:** 10.1371/journal.pone.0036718

**Published:** 2012-05-10

**Authors:** Haibing Chen, Zhi Zheng, Yan Huang, Kaifeng Guo, Junxi Lu, Lei Zhang, Haoyong Yu, Yuqian Bao, Weiping Jia

**Affiliations:** 1 Department of Endocrinology and Metabolism, Shanghai Clinical Center for Diabetes, Shanghai Jiaotong University Affiliated Sixth People's Hospital, Shanghai Diabetes Institute, Shanghai Key Laboratory of Diabetes Mellitus, Shanghai, China; 2 Department of Ophthalmology, First People's Hospital of Shanghai Affiliated to Shanghai Jiaotong University, Shanghai, China; University of Hong Kong, China

## Abstract

**Objective:**

To test the hypothesis that a microalbuminuria (MA) threshold can help predict the risk for the development of diabetic retinopathy (DR) in type 2 diabetes mellitus (T2DM)_ patients.

**Design:**

We conducted a cross-sectional study of 4739 subjects with T2DM and a prospective study of 297 subjects with T2DM in China respectively.

**Methods:**

Clinical and laboratory data were collected and biologic risk factors associated with any DR were analysed.

**Results:**

In the cross-sectional study, we found that MA was an independent risk factor for DR development; further, when the patients were divided into MA deciles, odds ratio (ORs) of DR for the patients in the sixth MA decile (10.7 mg/24 h) was 1.579-fold (1.161–2.147) compared to that for patients in the first MA decile. Furthermore, the OR of DR increased with a gradual increase in MA levels. Similarly, in the prospective study, during a mean follow-up of 4.5 years, we found that 51 patients (29.0%) of the 176 subjects with high MA level (10.7–30 mg/24 h) developed DR, while 17 patients (14.1%) of the 121 subjects with lower MA (<10.7 mg/24 h) developed DR, and the relative risk ratio of the development of DR is 2.13(95% CI, 1.58–3.62, *P*<0.001).

**Conclusion:**

These data suggest that an MA threshold can predict the risk for the development of DR in type 2 diabetes mellitus, although it is still within the traditionally established normal range.

## Introduction

Diabetic retinopathy (DR) is the most common microvascular complication of diabetes [1] and the leading cause of acquired blindness among people of working age in the Western world [2], [3]. The burden of DR is increasing with the rising prevalence of type 2 diabetes mellitus (DM) [4]. Despite major advances in the treatment of DR, affected subjects must be identified as early as possible by aggressively targeting the risk factors and by regular screening the affected individuals since timely intervention reduces DR progression and vision loss [5]. Numerous studies have shown that the risk factors for DR include HbA1c level, hypertension, dyslipidemia, duration of diabetes, age of onset, microalbuminuria (MA), and cigarette smoking [6], [7], [8], [9], [10].

MA seems to reflect a state of pathophysiological vascular dysfunction that makes an individual susceptible to organ damage [11]. Persistence of MA in diabetes patients is a risk marker not only for kidney and cardiac disorders, but also for severe ocular morbidity [12], [13], [14]. Numerous studies have reported that MA might be an independent risk factor for DR in type 1 and type 2 DM [12], [15], [16], [17]. On the other hand, Pedro RA reported that MA is a good predictor of DR in type 1 DM patients but not in type 2 DM patients (7). Therefore, we sought to clarify the role of MA in the development of retinopathy in Chinese patients with type 2 DM to determine if a natural MA threshold exists that can help determine the increased risk of DR, and, if so, to determine the optimal MA cut-off point for the identification of individuals at risk for DR development.

**Table 1:** Clinical characteristics of type 2 diabetes mellitus patients without or with retinopathy.

Variables	No DR	DR
Case (M/F)	3464 (1984/1480)	1275 (676/599)
Age (years)	58.6±13.9	60.6±11.2**
Duration of diabetes (years)	6.5±6.3	10.6±7.1**
Age of diagnosis	51.6±13.4	49.4±11.7**
SBP(mmHg)	130±17	136±19**
DBP(mmHg)	79±9	80±10**
Body-mass index(Kg/m2)	24.8±3.6	24.7±3.6
Waist circumference(cm)	88.4±10.6	88.5±10.6
HbA1c(%)	9.0±2.4	9.2±2.1**
FPG (mmol/l)	8.1±2.8	8.3±2.1
FCP ( ng/ml)	1.9±1.2	2.0±1.3
Antidiabetic drugs n(%)		
Monotherapy	983 (28.4%)	940 (27.1%)
Two-drug combinations	1604 (46.3%)	563 (44.2%)
≥Three-drug combinations	877 (25.3%)	328 (25.7)
Total cholesterol(mmol/l)	4.7±1.1	4.8±1.2
Low-density lipoprotein-cholesterol(mmol/l)	3.2±6.6	3.4±9.9
Triglyceride(mmol/l)	1.8±1.8	1.9±1.4
High density lipoprotein-cholesterol(mmol/l)	1.2±0.3	1.1±0.3
Antilipidemic drug		
Statins	347 (10%)	130 (10.2%)
Fibrats	102 (2.9%)	40 (3.1%)
Highly sensitive C-reactive protein(mg/l)	1.10 [0.51–2.82]	1.05 [0.44–2.53]
Microalbuminuria (mg/24h)	9.6 [3.3–209.8]	17.2 [4.0–591.8]**
eGFR(ml•min-1•1.73m2)	108±36	105±39**
Hypertention(%)	1478 (41.4%)	618(53.2%)**
Anti-hypertention medication		
RAS inhibitor (+)	451 (13.0%)	190 (14.9%)
DR(non/NPDR/PDR)	3464/0/0	0/1011/264

Data are expressed as the means ± SD or the geometric mean [95% confidence interval (CI)].

DR, diabetic retinopathy.

## Materials and Methods

The study was performed according to the principles of the Declaration of Helsinki and was approved by the local ethics committee. All study subjects provided informed consent.

A total of 4,739 patients (2,660 men and 2,079 women) were included in the cross-sectional study and 297 patients (132 men and 165 women) were recruited for the prospective study. All subjects were recruited from Shanghai Diabetes Centre at Shanghai Jiaotong University -Affiliated Sixth People's Hospital and Shanghai Ophthalmology Centre at Shanghai Jiaotong University -Affiliated First People's Hospital between the years 2005 to 2009. The diagnosis of diabetes mellitus was performed according to the World Health Organization (WHO) criteria which had been reported by WHO study group (1999). We excluded the patients with acute complications of diabetes mellitus, a history of non-diabetic renal disease, urinary tract infection, symptoms or history of a heart disease, and acute or severe chronic liver disease. In the prospective study, the data were collected both at baseline and at the patients' last visit assessment.

**Table 2:** Risk factors according to presence or absence of diabetic retinopathy as evaluated by a logistic regression model in 4,739 diabetes patients.

Variables	DR		
	Odds Ratio(95%CI)	p-value	β
Age (years)	0.986(0.981–0.992)	<0.001	−0.014
Sex	1.11(0.89–1.37)	0.342	
Duration of diabetes (years)	1.906(1.084–1.108)	<0.001	0.091
Age of diagnosis	0.98(0.98–1.012)	0.121	
SBP(mmHg)	1.013(1.008–1.017)	<0.001	0.012
DBP(mmHg)	0.972(0.961–1.015)	0.462	
Body-mass index(Kg/m2)	1.014(0.945–1.042)	0.722	
Waist circumference(cm)	0.988(0.980–0.996)	0.003	−0.012
HbA1c(%)	1.048(1.015–1.081)	0.004	0.046
Total cholesterol(mmol/l)	1.163(0.92–1.39)	0.332	
Low-density lipoprotein-cholesterol(mmol/l)	0.69(0.597–1.194)	0.124	
Triglyceride(mmol/l)	0.823(0.756–1.164)	0.132	
High density lipoprotein-cholesterol(mmol/l)	0.79(0.52–1.32)	0.273	
Highly sensitive C-reactive protein(mg/l)	0.93(0.88–1.02)	0.524	
Microalbuminuria(mg/24h)	1.163(1.134–1.193)	<0.001	0.151
eGFR(ml•min-1•1.73m2)	0.993(0.992–1.012)	0.282	
Hypertension (%)	1.05(0.78–1.37)	0.651	

Analysis of maximum likelihood estimates and stepwise selection of parameters.

Microalbuminuria were calculated as ranked variables from deciles of microalbuminuria.

DR, diabetic retinopathy.

Demographic and clinical data, including age, sex, duration of diabetes, weight, height, and medication, were recorded. Fasting blood samples were collected to determine fasting plasma glucose, fasting C peptide, HbA1c, serum lipid and lipoprotein, and serum creatinine levels. Serum concentrations of high-sensitivity C-reactive protein were measured using a Dade Behring Nephelometer II System (antiserum to human albumin; Siemens Healthcare Diagnostics). Albumin excretion rate (AER) was determined in 3 consecutive 24-h urine samples by using the Dade Behring Nephelometer II System, and the estimated glomerular filtration rate (eGFR) was calculated using the Modification of Diet in Renal Disease study equation [18]. Retinopathy status was assessed by fundus photography (TRC-NW100 camera; Nikon, Japan), and all images were graded by an experienced ophthalmologist. Presence of retinopathy was scored as nil, simplex, or proliferative.

**Table 3:** Microalbuminuria Deciles as Risk Factors for diabetic retinopathy in 4,739 diabetes patients.

Microalbuminuria Decile (mg/24 h)	DR	
	Odds Ratio(95%CI)	P value
<4.36	1 reference	–
≥4.36 to <5.63	1.159(0.844–1.593)	0.326
≥5.63 to <6.28	1.025(0.741–1.419)	0.88
≥6.28 to <8.49	1.186(0.863–1.629)	0.293
≥8.49 to <10.72	1.045 (0.723–1.512)	0.841
≥10.72 to <14.41	1.579(1.161–2.147)	0.004
≥14.41 to <21.43	2.053(1.521–2.772)	<0.001
≥21.43 to <43.0267	2.553(1.899–3.432)	<0.001
≥43.94 to <137.52	3.033(2.294–4.232)	<0.001
≥137.52	4.899(3.660–6.557)	<0.001

DR, diabetic retinopathy.

**Table 4:** Clinical characteristics of type 2 diabetic patients without retinopathy at baseline in prospective study.

Variables	Microalbuminuria< 10.72mg/24h	Microalbuminuria10.72–30mg/24h
Case (M/F)	121(53/68)	176 (79/97)
Age (years)	57.2±13.7	58.5±13.3*
Duration of diabetes (years)	6.2±6.0	6.6±6.2**
Age of diagnosis	51.2±12.7	52.0±12.4
SBP(mmHg)	126.6±15.6	131.6±15.4**
DBP(mmHg)	78.1±8.9	80.2±9.3**
Body-mass index(Kg/m2)	24.2±3.3	25.1±3.8**
Waist circumference(cm)	86.7±10.1	89.1±10.5**
HbA1c(%)	8.9±2.5	9.2±2.4**
Total cholesterol(mmol/l)	4.7±1.1	4.7±1.1
Low-density lipoprotein-cholesterol(mmol/l)	3.2±1.0	3.1±1.0
Triglyceride(mmol/l)	1.7±1.6	2.0±2.0**
High density lipoprotein-cholesterol(mmol/l)	1.1±0.3	1.2±0.3**
Highly sensitive C-reactive protein(mg/l)	0.92 [0.43–2.09]	1.29 [0.57–3.01]**
Microalbuminuria (mg/24h)	6.3±2.2	17.1±5.2**
eGFR(ml•min-1•1.73m2)	110.5±34.9	119.6±36.0
Hypertention(%)	40 (33.1%)	72(40.9%)**
Developed DR	17(14.0%)	51(28.98%)**

For normally distributed variables, the data are expressed as mean ± SD. For non-normally distributed values, data are expressed as the geometric mean [95% confidence interval (CI)]. The level of significance was determined using the *t* test for normally distributed values, and the Mann-Whitney *U* test was used for non-normally distributed values.

The relative contribution of covariates to DR risk was analyzed by logistic regression and stepwise selection of parameters. Odds ratios (ORs) for individual risk factors were calculated using logistic regression analyses. In multiple logistic regression analyses, DR was used as the dependent variable, and different risk factors which were identified in univariable logistic regression analysis were used as independent variables. The results are presented as OR with 95% CI.

All calculations were performed using the statistical package for social sciences (SPSS) 17.0 software (Los Angeles, CA). All reported *P* values were 2-tailed, and a P value of <0.05 was considered statistically significant.

## Results

### Difference in clinical characteristics between patients with DR and patients without DR

The demographics of the type 2 DM patients are shown in Table 1. Of the 4,739 DM patients that were recruited for this study, retinopathy occurred in 1,275 patients (26.9%) and nephropathy occurred in 1154 patients (24.4%). Retinopathy occurred in 794 patients (22.1%), with normoalbuminuria, 304 patients (35.6%) with MA, and 117 patients (59.0%) with macroalbuminuria. In patients with DR and without DR, there were significant differences in age (60.6±11.2 vs. 58.6±3.9 years, *P*<0.01), age of onset of diabetes (49.4±11.7 vs. 51.6±13.4 years, *P*<0.01), duration of diabetes (10.6±7.1 vs. 6.5±6.3 years, *P*<0.01), systolic blood pressure (SBP) (136±19 vs. 130±17 mmHg, *P*<0.001), diastolic blood pressure (80±9 vs. 79±10 mmHg, *P*<0.01), MA (17.2 [4.0–591.8] vs. 9.6 [5.87–21.2] mg/24 h, *P*<0.01), HbA1c level (9.2±2.1 vs. 9.0±2.4 %, *P*<0.01), eGFR (105±39 vs. 108±36 mL·min^−1^·1.73 m^2^, *P*<0.01), and the presence of diabetes (41.4% vs. 53.2%, *P*<0.01). There is no difference between the two groups in BMI, WC, FPG, FCP, lipids, and anti-diabetic medication, anti-hypertension medication.

### MA threshold to predict the risk for the development of DR

To determine the independent risk factors for DR, we performed logistic regression analysis. DR was used as the dependent variable. Different risk factors which were identified in univariable logistic regression analysis were used as independent variables. Our results showed that age, diabetes duration, SBP, waist circumference, HbA1c level, and MA were the 6 independent risk factors for DR (Table 2).

Further, to stratify the risk for DR, the patients were divided into MA deciles, with 472–475 patients in each group. The OR of DR for patients in the sixth MA decile (10.7 mg/24 h) was 1.579-fold (1.161–2.147) compared to that of patients in the first MA decile (P = 0.004). Furthermore, the OR of DR increased with a gradual increase in MA, and OR of DR for patients in the tenth MA decile was 4.899-fold (3.660–6.557) (P**<**0.001) (Table 3).

### A threshold effect for discriminating those with risk of DR was confirmed by a prospective study

A hundred seventy-six patients with MA 10.7∼30 mg/24 h (group 1) and 121 patients with MA<10.7 mg/24 h (group 2) were identified for the prospective study with a mean follow-up period of 4.5 years (range, 3.7–5.1 years). The clinical characteristics of the patients in the 2 groups are described in Table 4. Firstly, follow-up revealed that 68 patients (22.9%) developed DR. Secondly, this study further showed that 51 patients (29.0%) with higher MA levels (10.7∼30 mg/24 h) and 17 patients (14.1%) with lower MA levels (<10.71 mg/24 h) developed DR, and that there was a significant difference for prevalence of DR between the 2 groups (29.0% vs 14.1%, *P*<0.001). Notably, these results were largely unaffected by adjustments for age, creatinine clearance, hypertension, diabetes, and both total and high-density lipoprotein.

To further clarify the relationship between the development of DR and MA, development of DR was selected as a dependent variable, in contrast, MA as well as other clinical parameters was regarded as the independent variables to build a multiple logistic regression model. Only variables that significantly (p<0.05) differed from the developed DR and non-developed DR groups entered into the multiple logistic regression analysis. We found that MA≥10.7 mg/24 h was the independent factor for the development of DR (RR 2.13; 95% CI, 1.58–3.62), and the relationship existed even after the adjustment of other risk factors.

## Discussion

Epidemiological studies have shown that DR and nephropathy are closely associated [19] and that this correlation can be explained by a common mechanism involving tissue damage by the factors mentioned above such as HbA1c level, hypertension, dyslipidemia, duration of diabetes, age of onset, and cigarette smoking [6], [7], [8], [9], [10]. In 2007, the Kidney Disease Outcomes Quality Initiative (KDOQI) Clinical Practice Guidelines and Clinical Practice Recommendations for Diabetes and Chronic Kidney Disease [20], [21] stated that, in most patients with diabetes, chronic kidney disease is attributable to diabetes if MA is present along with DR, suggesting that DR plays an important role in the diagnosis of diabetic nephropathy (DN). In contrast, a precise role for MA in the screening for and monitoring of DR remains to be determined.

In the present study, we found that in diabetes patients with normoalbuminuria, microalbuminuria, and macroalbuminuria, the prevalence of DR was 22.1%, 35.6%, and 59.0%, respectively, which is similar to that reported in a previous study [22]. Logistic regression analysis revealed that MA was closely associated with DR, along with age, duration of diabetes, SBP, WC, and HbA1c, and these results were consistent with those of other studies [6], [7], [8], [9], [10], [19]. Further, when the patients were divided into MA deciles, we found that risk of DR for patients in the sixth MA decile (10.7 mg/24 h) was 1.579-fold (1.161–2.147) compared with that for the patients in the first MA decile. Furthermore, the risk of DR increased with albuminuria progression.

The predictive value of a high normal albuminuria for DN in patients with type 1 and type 2 DM has already been described [23], [24]. Recently, a study reported a 3-fold increased risk for DN in patients with urinary albumin excretion (UAE) levels above the median of 5 µg/min, and even higher progression rates for those with levels above 10 µg/min [25]. As stated above, in the present study, an MA threshold of 10.7 mg/24 h predicted increased risk for the development of DR in type 2 DM patients. There are several plausible explanations for the risk associated with normoalbuminuria: Caramori ML reported that normoalbuminuria does not imply normal renal function, and that long-standing normoalbuminuric type 1 DM patients have reduced GFRs that are associated with more advanced diabetic glomerular lesions and, probably, increased risk of progression [26]. Similarly, another study showed a significant association between DR and preclinical morphologic changes of DN in type 1 DM patients [27]. A similar situation may also exist in type 2 DM patients. In addition, we cannot rule out the possibility that conventional immunoassays routinely used for albuminuria measurement might underestimate the actual total urinary albumin levels, which includes immuno-unreactive albuminuria [28]. Indeed, the cut-off point is a rather arbitrary threshold based on findings from studies in the 1980s in which the progression rate to overt nephropathy in patients with values for albuminuria of 30 mg/24 h was about 80% [23].

One strength of our data is that a prospective study was carried out on the basis of the findings of the cross-sectional study. We found that the prevalence of DR in the patients with higher MA (≥10.7 mg/24 h) was as two times as that in the patients with lower MA (<10.7 mg/24 h) (29.0% vs 14.1%, *P*<0.001), and that there was a significant difference between the 2 groups, suggesting that a MA threshold of 10.7 mg/24 h could predict the risk for the development of DR in type 2 DM patients. We consider this supporting evidence that what we have observed is a true phenomenon. In this prospective study, several limitations should be acknowledged such as a small sample size and a short follow-up time.

It is interesting to note that, apart from its effects on vision, the presence of DR also signifies an increased risk of life-threatening systemic vascular complications, including stroke, coronary heart disease, heart failure, neuropathy, and nephropathy [29], [30], [31], [32]. Therefore, there is a need for redefining MA accordingly, in order to screen and monitor not only DR but also other microvascular and macrovascular complications. As reported by Klausen K, very low levels of MA (UAE level, 4.8 µg/min) are associated with an increased risk of coronary heart disease and death independent of renal function, hypertension, and diabetes. Klausen K also suggested that MA be redefined accordingly and that intervention studies be performed [14].

In conclusion, an MA threshold can predict the risk for the development of DR in type 2 diabetes mellitus, although it is still within the traditionally established normal range.

## References

[1] Cheung N, Mitchell P, Wong TY (2010). Diabetic retinopathy.. Lancet.

[2] Taylor HR, Keeffe JE (2001). World blindness: a 21st century perspective.. Br J Ophthalmol.

[3] Mohamed Q, Gillies MC, Wong TY (2007). Management of diabetic retinopathy: a systematic review.. Jama.

[4] Yang W, Lu J, Weng J, Jia W, Ji L (2010). Prevalence of diabetes among men and women in China.. N Engl J Med.

[5] Macky TA, Khater N, Al-Zamil MA, El Fishawy H, Soliman MM (2011). Epidemiology of diabetic retinopathy in Egypt: a hospital-based study.. Ophthalmic Res.

[6] West SK, Munoz B, Klein R, Broman AT, Sanchez R (2002). Risk factors for Type II diabetes and diabetic retinopathy in a mexican-american population: Proyecto VER.. Am J Ophthalmol.

[7] Wong TY, Cheung N, Tay WT, Wang JJ, Aung T (2008). Prevalence and risk factors for diabetic retinopathy: the Singapore Malay Eye Study.. Ophthalmology.

[8] Namperumalsamy P, Kim R, Vignesh TP, Nithya N, Royes J (2009). Prevalence and risk factors for diabetic retinopathy: a population-based assessment from Theni District, south India.. Br J Ophthalmol.

[9] Pedro RA, Ramon SA, Marc BB, Juan FB, Isabel MM (2010). Prevalence and relationship between diabetic retinopathy and nephropathy, and its risk factors in the North-East of Spain, a population-based study.. Ophthalmic Epidemiol.

[10] Wong J, Molyneaux L, Constantino M, Twigg SM, Yue DK (2008). Timing is everything: age of onset influences long-term retinopathy risk in type 2 diabetes, independent of traditional risk factors.. Diabetes Care.

[11] de Zeeuw D, Parving HH, Henning RH (2006). Microalbuminuria as an early marker for cardiovascular disease.. J Am Soc Nephrol.

[12] Thomas GN, Lin JW, Lam WW, Tomlinson B, Yeung V (2004). Albuminuria is a marker of increasing intracranial and extracranial vascular involvement in Type 2 diabetic Chinese patients.. Diabetologia.

[13] Deckert T, Yokoyama H, Mathiesen E, Ronn B, Jensen T (1996). Cohort study of predictive value of urinary albumin excretion for atherosclerotic vascular disease in patients with insulin dependent diabetes.. Bmj.

[14] Klausen K, Borch-Johnsen K, Feldt-Rasmussen B, Jensen G, Clausen P (2004). Very low levels of microalbuminuria are associated with increased risk of coronary heart disease and death independently of renal function, hypertension, and diabetes.. Circulation.

[15] (2000). Retinopathy and nephropathy in patients with type 1 diabetes four years after a trial of intensive therapy. The Diabetes Control and Complications Trial/Epidemiology of Diabetes Interventions and Complications Research Group.. N Engl J Med.

[16] Stratton IM, Kohner EM, Aldington SJ, Turner RC, Holman RR (2001). UKPDS 50: risk factors for incidence and progression of retinopathy in Type II diabetes over 6 years from diagnosis.. Diabetologia.

[17] Manaviat MR, Afkhami M, Shoja MR (2004). Retinopathy and microalbuminuria in type II diabetic patients.. BMC Ophthalmol.

[18] Levey AS, Bosch JP, Lewis JB, Greene T, Rogers N (1999). A more accurate method to estimate glomerular filtration rate from serum creatinine: a new prediction equation. Modification of Diet in Renal Disease Study Group.. Ann Intern Med.

[19] Knowler WC, Bennett PH, Ballintine EJ (1980). Increased incidence of retinopathy in diabetics with elevated blood pressure. A six-year follow-up study in Pima Indians.. N Engl J Med.

[20] Remuzzi G, Schieppati A, Ruggenenti P (2002). Clinical practice. Nephropathy in patients with type 2 diabetes.. N Engl J Med.

[21] (2007). KDOQI Clinical Practice Guidelines and Clinical Practice Recommendations for Diabetes and Chronic Kidney Disease.. Am J Kidney Dis.

[22] Girach A, Vignati L (2006). Diabetic microvascular complications--can the presence of one predict the development of another?. J Diabetes Complications.

[23] Gall MA, Hougaard P, Borch-Johnsen K, Parving HH (1997). Risk factors for development of incipient and overt diabetic nephropathy in patients with non-insulin dependent diabetes mellitus: prospective, observational study.. Bmj.

[24] Forsblom CM, Groop PH, Ekstrand A, Totterman KJ, Sane T (1998). Predictors of progression from normoalbuminuria to microalbuminuria in NIDDM.. Diabetes Care.

[25] Murussi M, Campagnolo N, Beck MO, Gross JL, Silveiro SP (2007). High-normal levels of albuminuria predict the development of micro- and macroalbuminuria and increased mortality in Brazilian Type 2 diabetic patients: an 8-year follow-up study.. Diabet Med.

[26] Caramori ML, Fioretto P, Mauer M (2003). Low glomerular filtration rate in normoalbuminuric type 1 diabetic patients: an indicator of more advanced glomerular lesions.. Diabetes.

[27] Klein R, Zinman B, Gardiner R, Suissa S, Donnelly SM (2005). The relationship of diabetic retinopathy to preclinical diabetic glomerulopathy lesions in type 1 diabetic patients: the Renin-Angiotensin System Study.. Diabetes.

[28] Comper WD, Osicka TM, Clark M, MacIsaac RJ, Jerums G (2004). Earlier detection of microalbuminuria in diabetic patients using a new urinary albumin assay.. Kidney Int.

[29] Cheung N, Rogers S, Couper DJ, Klein R, Sharrett AR (2007). Is diabetic retinopathy an independent risk factor for ischemic stroke?. Stroke.

[30] Cheung N, Wang JJ, Klein R, Couper DJ, Sharrett AR (2007). Diabetic retinopathy and the risk of coronary heart disease: the Atherosclerosis Risk in Communities Study.. Diabetes Care.

[31] Cheung N, Wang JJ, Rogers SL, Brancati F, Klein R (2008). Diabetic retinopathy and risk of heart failure.. J Am Coll Cardiol.

[32] Grauslund J, Green A, Sjolie AK (2008). Proliferative retinopathy and proteinuria predictmortality rate in type 1 diabetic patients from Fyn County, Denmark.. Diabetologia.

